# Ena/VASP Protein-Mediated Actin Polymerization Contributes to Naïve CD8^+^ T Cell Activation and Expansion by Promoting T Cell–APC Interactions *In Vivo*

**DOI:** 10.3389/fimmu.2022.856977

**Published:** 2022-06-09

**Authors:** Monique M. Waldman, Jeremy T. Rahkola, Ashton L. Sigler, Jeffrey W. Chung, Benjamin A. S. Willett, Ross M. Kedl, Rachel S. Friedman, Jordan Jacobelli

**Affiliations:** ^1^Department of Immunology and Microbiology, University of Colorado Anschutz Medical Campus, Aurora, CO, United States; ^2^Barbara Davis Research Center, University of Colorado Anschutz Medical Campus, Aurora, CO, United States; ^3^Rocky Mountain Regional Veterans Affairs (VA) Medical Center, Department of Medicine, University of Colorado Anschutz Medical Campus, Aurora, CO, United States; ^4^Department of Immunology and Genomic Medicine, National Jewish Health, Denver, CO, United States

**Keywords:** T cell, cytoskeleton, VASP, T cell activation, T cell motility, two-photon microscopy, immunological synapse, EVL

## Abstract

Naïve T cell activation in secondary lymphoid organs such as lymph nodes (LNs) occurs upon recognition of cognate antigen presented by antigen presenting cells (APCs). T cell activation requires cytoskeleton rearrangement and sustained interactions with APCs. Enabled/vasodilator-stimulated phosphoprotein (Ena/VASP) proteins are a family of cytoskeletal effector proteins responsible for actin polymerization and are frequently found at the leading edge of motile cells. Ena/VASP proteins have been implicated in motility and adhesion in various cell types, but their role in primary T cell interstitial motility and activation has not been explored. Our goal was to determine the contribution of Ena/VASP proteins to T cell–APC interactions, T cell activation, and T cell expansion *in vivo*. Our results showed that naïve T cells from Ena/VASP-deficient mice have a significant reduction in antigen-specific T cell accumulation following *Listeria monocytogenes* infection. The kinetics of T cell expansion impairment were further confirmed in Ena/VASP-deficient T cells stimulated *via* dendritic cell immunization. To investigate the cause of this T cell expansion defect, we analyzed T cell–APC interactions *in vivo* by two-photon microscopy and observed fewer Ena/VASP-deficient naïve T cells interacting with APCs in LNs during priming. We also determined that Ena/VASP-deficient T cells formed conjugates with significantly less actin polymerization at the T cell–APC synapse, and that these conjugates were less stable than their WT counterparts. Finally, we found that Ena/VASP-deficient T cells have less LFA-1 polarized to the T cell–APC synapse. Thus, we conclude that Ena/VASP proteins contribute to T cell actin remodeling during T cell–APC interactions, which promotes the initiation of stable T cell conjugates during APC scanning. Therefore, Ena/VASP proteins are required for efficient activation and expansion of T cells *in vivo*.

## Introduction

T cells patrol the body for signs of infection and cancer by recirculating through the blood and lymph and homing to secondary lymphoid organs (SLOs) such as lymph nodes (LNs). While patrolling, T cells scan antigen presenting cells (APCs) for the presence of their cognate peptide antigens bound to proteins encoded in the major histocompatibility complex (MHC). To scan for antigen within the LN, migrating T cells utilize the fibroblastic reticular cell (FRC) network and the associated network of lymphoid resident dendritic cells (DCs) as guiding structures for otherwise stochastic exploration (1–3). Naïve T cell motility is driven by chemokine receptor signaling, which stimulates actin polymerization. This actin polymerization is then translated into forward movement by the frictional interface mediated by low affinity integrin adhesion (4) and through interactions with the local environmental topography (5). T cells undergo rapid, amoeboid movement within LNs, and significant alterations in T cell speed and directionality can skew APC scanning and lead to defects in T cell priming (6, 7). Indeed, actin retrograde flow promoted by CCL19 enhances the crawling/scanning behavior of naive T cells and optimizes interactions with APCs, leading to a higher frequency of T cell encounters with rare cognate antigen *in vitro (* 8).

Upon encounter with cognate antigen-bearing APCs, T cells receive “stop signals” by which the T cell decelerates and eventually arrests as actin polymerization is translated towards the development of a stable interaction between the T cell and APC instead of into forward motility (9, 10). This can occur gradually and sequentially as T cells accumulate activation signals over multiple short serial interactions with APCs (11, 12). These serial interactions function to initiate the process of T cell activation, accumulating signals that lead to changes within the T cell necessary to override the pull of chemokine mediated motility. For example, T cell receptor (TCR) signaling leads to the downregulation of CCR7 and the conversion of LFA-1 into a high affinity conformation (13). This decreases chemokine mediated motility and increases adhesion between T cells and APCs, facilitating the transition into long-lasting and stable interactions.

To maintain sustained T cell–APC interactions, T cells undergo extensive actin reorganization and form an immunological synapse (IS). The IS is comprised of distinct zones of filamentous actin (F-actin) composition, which also structurally facilitates the organization, movement, and internalization of TCRs and co-receptors (14–16). Within these synapses, microclusters of TCRs, co-stimulatory molecules, and adhesion receptors engage their ligands on the APC surface to trigger downstream signaling, resulting in T cell activation and proliferation (17). Actin cytoskeletal effectors, such as formins and WASP, have been shown to contribute to the formation of actin structures important for TCR centralization and to actin retrograde flow important for regulating integrin function and T cell–APC interactions (18–23). The classic, TCR monofocal configuration of the IS is typically observed in artificial synapses created with supported lipid bilayers or in synapses with APC cell lines (24, 25). However, physiological interactions between T cells and DCs often lead to multifocal synapses, in which there are multiple local ensembles of TCR, co-receptors, and adhesion molecules (26). While there have been many effector molecules identified as essential for these processes in *in vitro* systems, there is still more to uncover in detailing the specific proteins responsible for actin rearrangement throughout T cell activation. In particular, the function of specific cytoskeletal effectors of actin remodeling in primary T cell activation *in vivo* is still mostly unknown.

The spatial and temporal organization of the actin cytoskeleton is regulated at many levels: cells rely on effectors of actin polymerization to efficiently nucleate and elongate actin filaments, actin filament capping proteins to stop polymerization, and severing proteins to break filaments (27, 28). Branched actin networks are initiated through the actin-related protein 2/3 (Arp2/3) complex, which binds to pre-existing actin filaments to nucleate new filaments at an angle of 70 degrees. In T cells, linear actin polymerization is conducted by two major families of effector proteins: the formin family and the enabled/vasodilator-stimulated phosphoprotein (Ena/VASP) family. The formin family, which can both nucleate new linear actin filament production and elongate actin filaments, has been implicated in T cell activation, egress from the thymus, and activated T cell trafficking to inflamed tissues (29–32). Ena/VASP proteins enhance barbed-end elongation and prevent capping proteins from binding. Although Ena/VASP proteins are not actin polymerization nucleators, they participate in forming branched actin networks by linearly elongating filaments nucleated by Arp2/3, and in linear actin networks by elongating formin-initiated filaments (33, 34). The Ena/VASP family is composed of three members—Mena (mammalian Ena), Ena/VASP-like (EVL), and VASP—though only EVL and VASP are expressed in hematopoietic cells (35). EVL and VASP share significant structural homology including an N-terminal EVH1 domain, which regulates cellular localization, and a C-terminal EVH2 domain, which mediates interactions with actin (36). EVL and VASP localize to lamellipodia, filopodia tips, and adhesive sites such as fibroblast focal adhesions (36–43). Fibroblasts lacking Ena/VASP produce shorter filopodia and a slower moving lamellipodium, which paradoxically leads to enhanced fibroblast motility (44). In other cell types, however, loss of Ena/VASP impairs the generation of traction forces as well as integrin-mediated adhesion and can impair motility (45). Further, metastatic cancers express higher levels of EVL, and small-molecule inhibitors of the EVH1 domain impair invasion and extravasation of breast cancer cells (46, 47).

While the roles of the Ena/VASP protein family in the motility, adhesion, and sensory capacity of many cell types are well defined, they have not been studied extensively in T cells. Data from experiments using Jurkat cells *in vitro* suggests that EVL plays a role in actin remodeling downstream of TCR signaling (40, 48), but this has not been confirmed in primary cells or *in vivo*. Previous work from our lab indicates that EVL and VASP play a role in the expression and function of the integrin alpha-4 subunit (CD49d), and are therefore required for activated T cell transendothelial migration and trafficking, but are not necessary for naïve T cell trafficking to LNs and spleen (49). This previous work also showed that EVL and VASP can compensate for each other since only EVL/VASP doubly deficient T cells show a trafficking defect (49).

Given the roles of the Ena/VASP protein family in other cell types, coupled with the understanding that actin remodeling has key effects on T cell activation, we sought to explore the role of the Ena/VASP protein family in the cytoskeletal rearrangements necessary for T cell navigation in LNs, finding and engaging APCs, and activation *in vivo*. In this paper, we demonstrate that EVL/VASP proteins contribute to the accumulation of T cells during an immune response. Further, we find a novel role for the Ena/VASP protein family in facilitating the initiation and stability of T cell–APC interactions, and in mediating actin polymerization at the IS.

## Results

### Ena/VASP Deficiency Reduces T Cell Expansion in Response to *Listeria monocytogenes* Challenge

**Figure 1:**
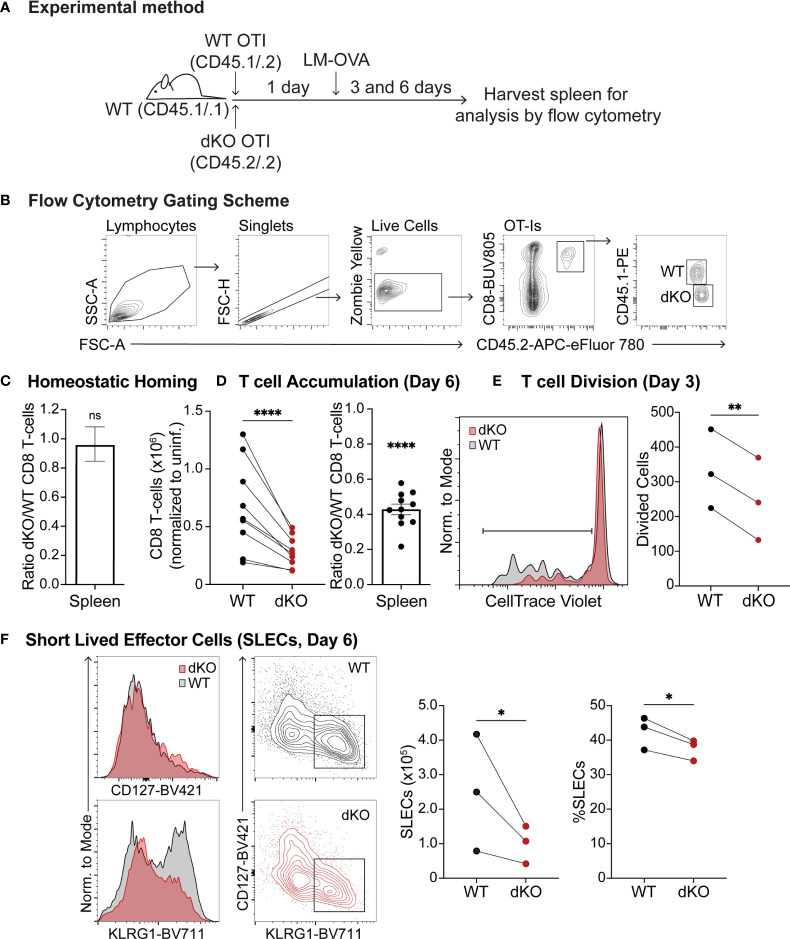
Ena/VASP deficiency reduces T cell accumulation in response to Listeria Monocytogenes challenge. Congenically distinct naïve WT CD45^.1/.2^ OT-I and EVL/VASP dKO CD45^.2/.2^ OT-I T cells were isolated from donor mice and transferred at a 1:1 ratio I.V. into WT CD45^.1/.1^ recipient mice. **(A)** Graphical schematic of the experimental method. **(B)** Representative flow cytometry gating scheme used to detect transferred T cells. **(C)** WT and EVL/VASP dKO naïve T cells have similar persistence *in vivo*. 1×10^6^ WT and EVL/VASP dKO naïve OT-I CD8^+^ T cells, were co-transferred I.V. into uninfected WT CD45^.1/.1^ recipient mice and spleens were harvested 6 days later to determine a persistence baseline. **(D–F)** EVL/VASP dKO T cells have reduced proliferation and accumulation in response to LM challenge. 10,000 of each naïve WT CD45^.1/.2^ OT-I and EVL/VASP dKO CD45^.2/.2^ OT-I T cells were co-transferred I.V. into WT CD45^.1/.1^ recipient mice one day before infection with LM-OVA (2×10^3^ PFUs). Spleens from recipient mice were then harvested 3 or 6 days later for analysis by flow cytometry. **(D)** T cell numbers recovered from the spleen at day 6 post LM infection (left) and ratios of EVL/VASP dKO/WT CD8^+^ T cells (right). EVL/VASP dKO T cell numbers were normalized to the ratio of EVL/VASP dKO/WT T cells recovered from spleens of uninfected recipient mice at day 6 from the same experiment [i.e., homing ratio in **(C)**]. **(E)** Example CTV proliferation dye dilution curves and quantification of the average number of divided cells, determined by CTV dilution, from spleens on day 3 post LM-OVA infection. **(F)** Representative histograms of KLRG1 and CD127 expression, and flow cytometry contour plots showing the gates used to identify SLECs (KLRG1^hi^ CD127^lo^). The number (normalized to uninfected mice) and percent of WT and EVL/VASP dKO SLEC T cells are plotted. Data are from ≥9 experiments each with ≥2 mice per group/experiment, except for **(E)**, **(F)**, which are from 3 independent experiments with ≥2 mice per group/experiment. Significance was defined by paired t tests (for T cell numbers) and one sample t tests compared to a hypothetical value of 1.0 (for the EVL/VASP dKO/WT ratios); ns is not significant, * is p < 0.05, ** is p < 0.01, and **** is p < 0.0001.

Since EVL/VASP proteins can promote actin polymerization in response to CD3 stimulation in Jurkat T cells (48) and effector T cell trafficking and integrin function are modulated by Ena/VASP proteins (49), we investigated the role of Ena/VASP proteins in T cell responses and differentiation *in vivo*. We thus analyzed the effect of EVL/VASP deletion in naïve CD8^+^ T cell expansion following *Listeria monocytogenes* (LM) infection. To assess T cell intrinsic effects, we adoptively co-transferred low numbers (to approximate physiological conditions) of naïve WT and EVL/VASP double knockout (dKO) Ovalbumin (OVA)-specific CD8^+^ OT-I T cells into WT recipient mice (**Figures 1A, B**). First, we established that under homeostatic conditions naïve WT and EVL/VASP dKO OT-I T cells had similar homing and persistence in the spleen (**Figure 1C**). Next, we infected recipient mice with LM expressing OVA (LM-OVA). On day 6 after the infection, we found that the number of EVL/VASP dKO OT-I T cells in the spleen was significantly reduced compared to their WT counterparts (2.6-fold average reduction compared to WT) (**Figure 1D**). We next analyzed T cell proliferation on day 3 post-LM infection by proliferation dye dilution. Our data showed a significantly reduced number of divided T cells in the EVL/VASP dKO population (**Figure 1E**). These data suggest that EVL/VASP proteins play an important role in CD8^+^ T cell expansion and accumulation in response to LM infection. Additionally, in the EVL/VASP dKO population there was a small but significant reduction in the number and percentage of short-lived effector cells (SLECs), characterized by KLRG1^hi^ CD127^lo^ expression (**Figure 1F**).

### EVL/VASP dKO T Cells Defects Are not Due to Thymic Priming Differences

We previously showed that T cell subset development is largely normal in the EVL/VASP dKO mice (49). However, since EVL/VASP dKO mice are germline double knockouts, their thymic epithelial cells and DCs are also deficient in EVL/VASP, which may alter T cell signaling and priming during thymocyte development. Thus, to ensure that phenotypic differences observed in EVL/VASP dKO T cells were T cell-intrinsic and not due to T cell development in an EVL/VASP-deficient thymic environment, we created bone marrow chimeras. We transferred bone marrow from WT or EVL/VASP dKO OT-I mice into separate WT recipients, such that both control and EVL/VASP dKO T cells matured within WT thymuses. After ≥8 weeks, we isolated peripheral EVL/VASP dKO and WT OT-I T cells from the LNs and spleen of bone marrow chimeras and adoptively co-transferred them into WT recipient mice for LM-OVA infection (**Supplementary Figure 1A**). We then compared T cell numbers in the spleens 6 days after LM-OVA infection as described above. Regardless of whether T cells matured in bone marrow chimeras or endogenously in intact mice, EVL/VASP dKO T cell responses were similarly impaired compared to WT T cells (**Supplementary Figure 1B**). These results indicate that EVL/VASP dKO T cell expansion defects are T-cell intrinsic, and not due to differences in the thymic environment in which they develop.

### Ena/VASP Deficiency Reduces T Cell Expansion Following Immunization With Dendritic Cells

**Figure 2:**
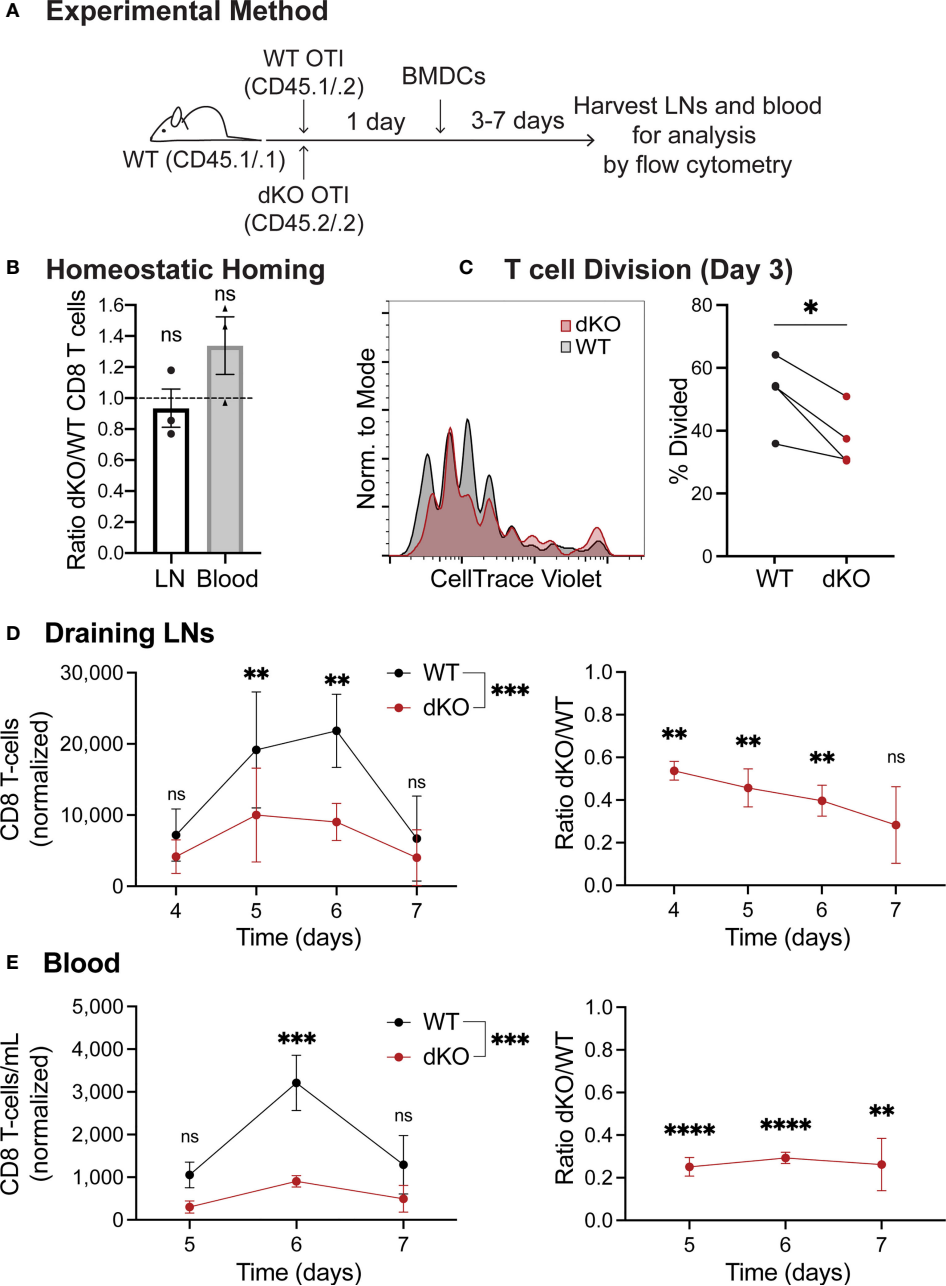
Ena/VASP deficiency reduces T cell expansion in response to stimulation with bone marrow-derived dendritic cells *in vivo*. Congenically distinct naïve WT CD45^.1/.2^ OT-I and EVL/VASP dKO CD45^.2/.2^ OT-I T cells were isolated from donor mice and co-transferred I.V. into WT CD45^.1/.1^ recipient mice. **(A)** Graphical schematic of the experimental method. **(B)** WT and EVL/VASP dKO T cells have similar homing to LNs and persistence in the blood. 1×10^6^ WT and EVL/VASP dKO naïve OT-I CD8^+^ T cells, were co-transferred I.V. into unimmunized WT CD45^.1/.1^ recipient mice and LNs and blood were harvested 24 hours later to determine a homing and persistence baseline. **(C–E)** EVL/VASP dKO T cells have reduced proliferation and expansion in response to BMDC stimulation. 10,000 of each naïve WT CD45^.1/.2^ OT-I and EVL/VASP dKO CD45^.2/.2^ OT-I T cells were co-transferred I.V. into WT CD45^.1/.1^ recipient mice. 24 hours later, 2.5×10^5^ mature, LPS-activated OVA-pulsed BMDCs were injected subcutaneously into both hind footpads of recipient mice. At the indicated time-points, mice were euthanized and the draining popliteal LNs and blood were collected for analysis by flow cytometry. **(C)** Reduced proliferation of EVL/VASP dKO T cells. Example CTV proliferation dye dilution curves and quantification of the average percent of T cells divided, 3 days post BMDC immunization. **(D, E)** Reduced expansion of EVL/VASP dKO T cells. T cell numbers in draining popliteal LNs and blood measured at days 4–7 after stimulation with BMDCs (left) and ratios of EVL/VASP dKO/WT T cells (right). EVL/VASP dKO T cell numbers were normalized to the ratio of EVL/VASP dKO/WT T cells recovered from the equivalent tissue of unimmunized recipient mice from the same experiment. Data shown are means ± SEM from ≥3 experiments with ≥2 mice per group. Significance was assessed by 2-way ANOVA (for T cell numbers) and one sample t test compared to a hypothetical value of 1.0 (for the EVL/VASP dKO/WT ratio); ns is not significant, * is p < 0.05, ** is p < 0.01, *** is p < 0.001, and **** is p < 0.0001.

To investigate the cause of the T cell expansion defect, we next established an *in vivo* system in which we could activate T cells while also being able to visualize the interaction of T cells with APCs in the physiological environment of a LN. For this purpose, we used antigen-pulsed bone marrow-derived dendritic cells (BMDCs) as a stimulus (**Figure 2A**). First, similar to the LM infection experiments, we confirmed that WT and EVL/VASP dKO T cells had comparable homing to LNs under homeostatic conditions in the absence of antigen stimulation (**Figure 2B**). We then analyzed T cell responses by subcutaneously injecting mature, LPS-activated, SIINFEKL peptide-pulsed BMDCs into mice bearing co-adoptively transferred WT and EVL/VASP dKO OT-I T cells (**Figure 2A**). We analyzed T cell proliferation 3 days post BMDC immunization and found a significant reduction in the percentage of divided EVL/VASP dKO T cells (**Figure 2C**). We also performed a time-course analysis and determined that this experimental system confirmed the T cell expansion defect seen with LM challenge. Specifically, upon BMDC immunization we detected significantly fewer EVL/VASP dKO T cells at the peak of the T cell effector response in draining LNs (Day 4–7: 1.9–3.5-fold less) as well as in the blood (Day 5–7: 3.4–4.0-fold less) once the activated T cells began exiting the LNs (**Figures 2D, E**). These differences in EVL/VASP dKO T cell numbers were also similar in highly vascularized organs such as the spleen, liver, and lungs (data not shown).

### EVL/VASP dKO Naïve T Cells Navigate the LN Normally Under Homeostatic Conditions

We then inquired if the T cell expansion defect was in part due to reduced ability of naïve EVL/VASP dKO T cells to migrate within lymph nodes and thus encounter APCs and be activated. Therefore, we compared EVL/VASP dKO and WT naïve T cell motility in the LN under homeostatic conditions in the absence of cognate antigen. Analysis of T cell motility in lymph nodes showed no significant differences in the speed, displacement, straightness, or arrest coefficient between WT and EVL/VASP dKO naïve T cells (**Supplementary Figure 2**, **Supplementary Movie 1**). This suggests that an inherent motility defect is not the cause of impaired T cell expansion of the EVL/VASP dKO naïve T cells.

### EVL/VASP dKO T Cells Have Impaired APC Interactions *In Vivo*

**Figure 3:**
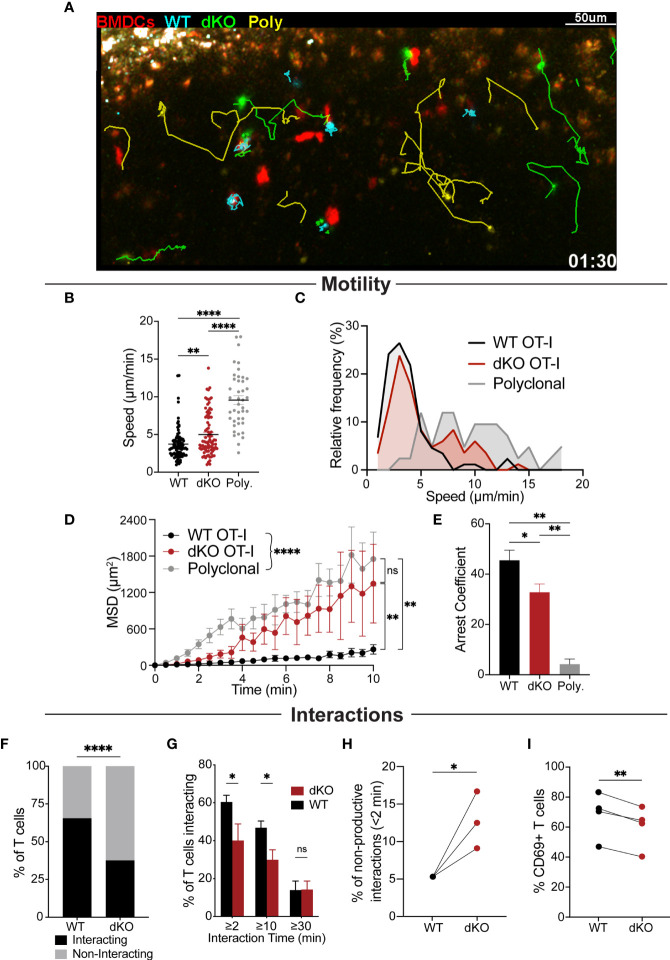
EVL/VASP dKO T cells have impaired APC interactions *in vivo*. Naïve WT OT-I and EVL/VASP dKO OT-I T cells were isolated from donor mice, differentially dye labeled with CTV or CTFR, and 20,000 of each were co-transferred into WT recipient mice. In a subset of experiments, 200,000 polyclonal WT T cells dye labelled with CFSE were also co-transferred with the OT-I T cells. 24 hours later, OVA-pulsed tdTomato-BMDCs were injected subcutaneously into recipient mice. After 13–19 hours, mice were euthanized and the draining popliteal LNs were harvested and analyzed by time-lapse two-photon microscopy. T cells were tracked and their interactions with BMDCs were also quantified. **(A)** Representative snapshot from a movie depicting the movement of EVL/VASP dKO OT-I T cells (green), WT OT-I T cells (cyan), polyclonal control T cells (yellow), and BMDCs (red). Track lines show the path of T cell movement imaged over 30 minutes. Scale bar, 50 µm. **(B)** Mean velocities of WT OT-I, EVL/VASP dKO OT-I, and polyclonal control T cells in the presence of antigen-pulsed BMDCs. Each dot represents a single T cell. **(C)** Frequency distribution of T cell speed. **(D)** Mean square displacement (MSD, +/-SEM) over time of WT OT-I, EVL/VASP dKO OT-I, and polyclonal control T cells. **(E)** Average arrest coefficient (percentage of time that a T cell’s instantaneous velocity <2 μm/min) of WT OT-I, EVL/VASP dKO OT-I, and polyclonal control T cells. Mean +/- SEM. **(F)** Total number of WT and EVL/VASP dKO OT-I T cells interacting (for at least 2 minutes) and non-interacting with APCs. n=130 WT cells and 128 EVL/VASP dKO cells. **(G)** Distribution of T cell–APC interaction times shown by graphing percent of WT and EVL/VASP dKO OT-I T cells interacting with BMDCs for given durations. **(H)** Quantification of non-productive T cell–APC interactions: graph of percent of interactions that occurred in which T cells disengaged from APCs within 2 minutes. **(I)** Quantification of CD69 expression in T cells from the LNs of the imaging experiments. Immediately following two-photon microscopy, imaged LNs were digested and analyzed by flow cytometry. Data in **(B–G)** are from ≥5 experiments each with ≥2 mice per group, data in **(H)** are from 3 experiments, as experiments with <20 cells were excluded, and data in **(I)** are from 4 experiments. Significance was assessed by 1-way ANOVA **(B, E)**, 2-way ANOVA **(D, G)**, paired t test **(H, I)**, and Fisher’s exact test **(F)**; ns is not significant, * is p < 0.05, ** is p < 0.01, and **** is p < 0.0001.

We next sought to assess whether EVL/VASP dKO T cell expansion defects originate during the T cell priming phase *in vivo*. For these experiments, we used fluorescent BMDCs to stimulate adoptively transferred differentially dye-labeled WT and EVL/VASP dKO OT-I T cells along with polyclonal T cell controls (experimental setup similar to that depicted in **Figure 2A**). While imaging experiments typically rely on high numbers (≥2×10^6^) of transferred T cells (1–3, 11, 12, 50–54), we maintained the more physiological low T cell numbers (2×10^4^) established in our *in vivo* T cell activation experiments. This required collecting a large amount of time-lapse data from multiple recipient mice in order to acquire a sufficient number of T cells for analysis, given the low precursor frequency of antigen-specific T cells used. We visualized T cell–APC interactions during the early stages of T cell activation *in vivo* by two-photon microscopy 13-19 hours post BMDC transfer (**Figure 3A** and **Supplementary Movies 2**, **3**). Previous work suggests that depending on the time-point and T cells analyzed, a variety of both transient and sustained interactions with DCs can be visualized (11, 12, 55). However, since data from *in vivo* imaging with physiologically low numbers of antigen-specific T cells is lacking, it was unknown what kind of T cell–APC interaction dynamics should be expected.

Quantification of T cell motility parameters in the presence of antigen-pulsed BMDCs indicated that EVL/VASP dKO OT-I T cells had higher average motility rates, greater displacement, and reduced arrest compared to WT OT-I T cells (**Figures 3B–E**), displaying an intermediate phenotype in-between WT antigen-specific and non-antigen-specific polyclonal T cells. These motility data also showed that EVL/VASP dKO cells decelerated less than WT cells, suggesting reduced frequency and/or duration of interactions with APCs. Indeed, in examining the frequency distribution of T cell speeds, we found that a subset of dKO cells had higher speeds, more similar to the polyclonal T cells, and this higher speed subset was practically absent from the antigen-specific WT T cell population (**Figure 3C**). The higher variability in the displacement of the EVL/VASP dKO population (**Figure 3D**) further suggests that these data likely represent a mixed population of dKO T cells—some interact with APCs while others do not. Thus, a subset of EVL/VASP dKO T cells exhibited motility patterns similar to the WT OT-I T cells while other EVL/VASP dKO T cells exhibited motility similar to the polyclonal T cell population. We next directly analyzed T cell–APC interactions in LNs. Our data showed that a greater percentage of WT OT-I T cells interacted for at least 2 minutes with BMDCs compared to EVL/VASP dKO OT-I T cells (**Figure 3F**). Additionally, significantly more WT OT-I T cells formed contacts with BMDCs that lasted for at least 10 minutes; however, the frequency of T cell–APC interactions longer than 30 minutes was similar for EVL/VASP dKO T cells (**Figure 3G**). Finally, we also found that of the T cells that did contact BMDCs, a greater percentage of T cell–APC interactions were non-productive (lasting less than 2 min) amongst EVL/VASP dKO T cells compared to their WT counterparts (**Figure 3H**). Together this suggests a defect in the ability of a subset of EVL/VASP dKO T cells to shift from an initial contact to a stable T cell–APC interaction. Overall, these motility and T cell–APC interaction data support that a driving factor leading to the difference between WT and EVL/VASP dKO OT-I T cell interactions with BMDCs is in their ability to stop migrating and form a stable interaction *in vivo*.

We next sought to gain a readout of the potential functional consequence of the impaired *in vivo* APC interactions of EVL/VASP dKO T cells during this early priming phase. Thus, immediately following two-photon microscopy, the imaged LNs were digested, and T cells were analyzed by flow cytometry to assess their activation status. Expression of the early T cell activation marker CD69 was significantly reduced in the EVL/VASP dKO T cells, consistent with the observed reduction in T cell–APC interactions (**Figure 3I**).

### EVL/VASP dKO T Cell Signaling and Proliferation *In Vitro* Are Normal

**Figure 4:**
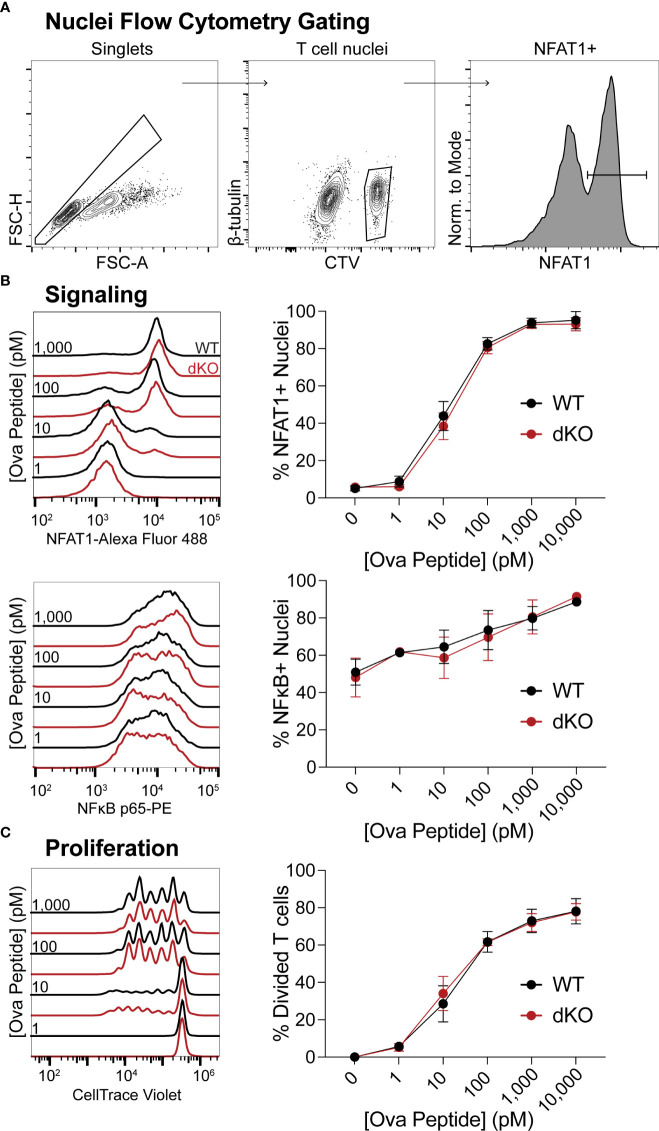
EVL/VASP-deficient T cell signaling and proliferation *in vitro* are normal. Naïve WT and EVL/VASP dKO OT-I T cells were CTV dye-labeled, mixed with antigen-pulsed WT splenocytes, centrifuged, and cocultured. **(A)** Representative flow cytometry gating scheme used to identify isolated T cell nuclei in **(B)**. **(B)** T cells were incubated with splenocytes for 45 minutes, and nuclei were isolated for analysis of nuclear translocation of transcription factors by flow cytometry. Left: Representative histograms of NFAT1 (top) or NFκB (bottom) fluorescence in WT and EVL/VASP dKO T cell nuclei. Right: Dose response plots of the percentage of NFAT1+ (top) or NFκB+ (bottom) T cell nuclei. **(C)** T cells were cocultured with SIINFEKL-pulsed splenocytes for 3 days, and proliferation was assessed by CTV dye dilution. Left: representative histograms of CTV dilution; right: dose response plot shows quantification of % of T cells divided. Data shown are means ± SEM; averaged from ≥3 experiments each with ≥3 replicates per group. There was no significant difference between WT and EVL/VASP dKO T cells, as assessed by two-way ANOVA interaction effects.

Having determined that EVL/VASP-deficient T cells have an expansion defect following *in vivo* pathogen challenge and reduced ability to form conjugates with APCs *in vivo*, we asked whether EVL/VASP dKO T cells have generalized impaired signaling and/or ability to proliferate. To assess whether the Ena/VASP family plays a role in the signaling required for T cell activation, we stimulated WT and EVL/VASP dKO OT-I T cells with WT splenocytes as APCs pulsed with varying concentrations of SIINFEKL peptide *in vitro*, and spun APCs and T cells together to enforce T cell–APC encounters. Then, to evaluate TCR signaling, nuclei from the T cell–APC conjugates were isolated and analyzed by flow cytometry to quantify translocation into the nucleus of transcription factors associated with T cell activation, NFAT1 and NFκB, as previously described (56) (**Figure 4A**). For both transcription factors, there was no difference in nucleus translocation between WT and EVL/VASP dKO T cells at various antigen concentrations (**Figure 4B**). To evaluate proliferation capacity, WT and EVL/VASP dKO OT-I T cells were stimulated *in vitro* with antigen-pulsed splenocytes and cultured for 3 days, and cell division was quantified by dilution of CellTrace Violet (CTV) proliferation dye. No difference between WT and EVL/VASP dKO T cell proliferation was detected in this setting (**Figure 4C**). Taken together, these results demonstrate that EVL/VASP dKO T cells do not have an inherent/intrinsic proliferation defect. Furthermore, in these *in vitro* conditions, EVL/VASP dKO T cell stimulation is also normal, indicating that Ena/VASP proteins are not required for TCR-mediated signaling leading to NFAT1 and NFκB translocation or proliferation.

### EVL/VASP dKO T Cells Have Impaired F-Actin Polarization at the Immune Synapse and Form Conjugates With APCs That Are Less Stable

**Figure 5:**
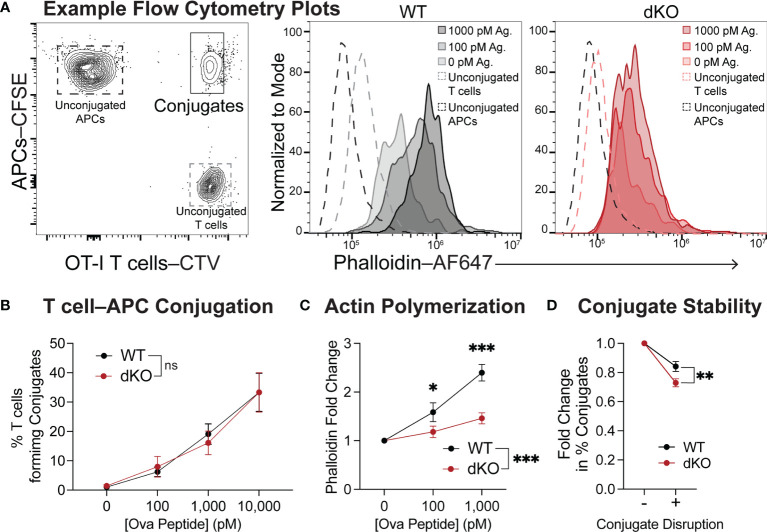
EVL/VASP-deficient T cells have impaired actin polymerization during conjugation with APCs and form fewer stable conjugates. Naïve WT and EVL/VASP dKO OT-I T cells were dye-labeled with CTV and mixed with CFSE-labeled WT splenocytes pulsed with SIINFEKL peptide. T cells and APCs were centrifuged together, cocultured for 2 minutes, and stained for F-actin with phalloidin. **(A)** Left; gating used to identify T cell–APC conjugates. Right; example histograms of phalloidin staining in the conjugates formed by WT and EVL/VASP dKO T cells. **(B)** Quantification of the % of naïve WT and EVL/VASP dKO OT-I T cells that formed conjugates with APCs. No significant difference (ns) determined by 2-way ANOVA. **(C)** F-actin polymerization in T cell–APC conjugates. Graph depicts the fold change in phalloidin (GMFI) in WT and EVL/VASP dKO T cell conjugates compared to conjugates that formed in the absence of antigen (0 pM). Data are from 3 experiments each with ≥2 replicates per group. Significance was assessed by 2-way ANOVA; * is p < 0.05, *** is p < 0.001. **(D)** T cell conjugate stability was quantified by comparing T cell–APC conjugates (using 1000 pM OVA peptide) fixed immediately after 2-minute incubation (- Conjugate Disruption) or disturbed by briefly vortexing tubes before fixation (+ Conjugate Disruption). Graph depicts fold change in % of conjugates from the undisrupted control. Data are from 3 experiments with 3 replicates per group. Significance was assessed by paired t test; ** is p < 0.01.

Our *in vivo* imaging data supports that EVL/VASP-deficient T cells have an impairment in establishing stable interactions with APCs. First, we tested whether there were any differences in the ability of EVL/VASP dKO T cells to form conjugates with APCs *in vitro* when the cells were spun together to facilitate contacts (**Figure 5A** left panel). At all concentrations of antigen tested, EVL/VASP dKO and WT OT-I T cells had similar conjugation efficiency *in vitro* (**Figure 5B**). Given that Ena/VASP proteins participate in actin network remodeling, we asked if EVL/VASP dKO T cells were impaired in actin polymerization upon encountering antigen-bearing APCs. Using the same *in vitro* setup for conjugate formation, we stimulated WT and EVL/VASP dKO OT-I T cells with splenocytes pulsed with varying concentrations of SIINFEKL peptide *in vitro*, and quantified actin polymerization in the T cell–APC conjugates. Using phalloidin to stain for F-actin we found a strong impairment in actin polymerization in the EVL/VASP dKO T cell–APC conjugates by flow cytometry (**Figures 5A, C**). To explore whether this impaired actin polymerization correlated with differences in stability of T cell–APC conjugates, we repeated the conjugate formation experiment, but disrupted the conjugates by vortexing to dissociate those with weak interactions before fixation. Indeed, the EVL/VASP dKO T cells lost slightly, but significantly, more conjugates after disruption than WT T cells, implying that the EVL/VASP dKO conjugates are less stable (**Figure 5D**).

**Figure 6:**
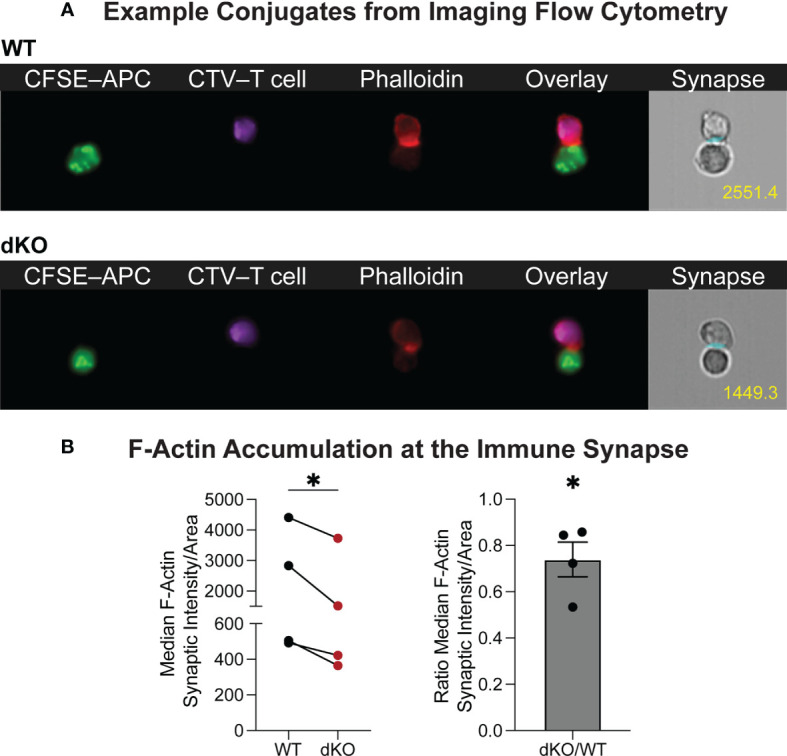
Ena/VASP proteins contribute to actin rearrangement at the immunological synapse. CTV-labeled WT or EVL/VASP dKO naïve OT-I T cells were conjugated with antigen-loaded CFSE-labeled APCs and stained for F-actin. **(A)** Representative ImageStream images of WT and EVL/VASP dKO T cell–APC conjugates stained for phalloidin. Number in yellow indicates phalloidin GMFI measured at the immune synapse. **(B)** Left; quantification of median phalloidin intensity at the immunological synapse divided by the synapse area. Right; quantification of the EVL/VASP dKO/WT fluorescence intensity ratio at the synapse. Data represent averages from 4 independent experiments with ~1000–7000 conjugates analyzed per sample. Statistics were performed using one-tailed paired t test and one sample t test compared to a hypothetical value of 1.0; * is p < 0.05.

**Figure 7:**
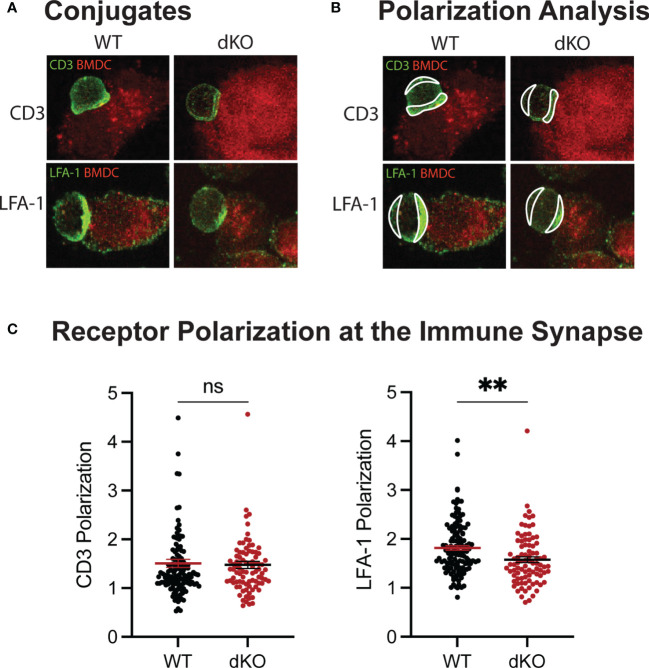
EVL/VASP dKO T cells polarize less LFA-1 at the immunological synapse. Mature, LPS-activated, OVA-pulsed tdTomato-BMDCs were adhered to chamber slides. Naïve WT and EVL/VASP dKO OT-I T cells were added to the chamber slides to form conjugates. Conjugates were then fixed and stained for CD3 and LFA-1. **(A)** Example images of conjugates stained for CD3 (top) or LFA-1 (bottom). **(B)** Examples of regions drawn to assess polarization of receptors at the immunological synapse. **(C)** Quantification of receptor polarization at the immune synapse. The mean fluorescence intensity (MFI) of the contact region at the synapse was divided by the MFI of the region defined at the back of the cell. Each dot represents an individual T cell–APC conjugate, data are pooled from 3 independent experiments. Significance was assessed by unpaired t test; ns is not significant, and ** is p < 0.01.

Furthermore, using the ImageStream platform for high throughput imaging of T cell–APC conjugates, we analyzed F-actin specifically at the immune synapse of WT and EVL/VASP dKO OT-I T cells. Our data show a reduction in the level of actin polymerization at the T cell–APC interface formed by EVL/VASP dKO T cells compared to their WT counterparts (**Figure 6**). This observation suggests that EVL/VASP proteins play a role in actin polymerization at the IS during T cell–APC interactions. To garner further insight into how T cell–APC conjugate stability may be modulated by Ena/VASP proteins, we analyzed the accumulation of the TCR complex and LFA-1 integrin at the IS in WT and EVL/VASP dKO OT-I T cells (**Figures 7A, B**). While our data showed equivalent CD3 accumulation at the synapse, we found a small but significant reduction in the amount of LFA-1 that polarized to the T cell–APC interface (**Figure 7C**). Overall, these data suggest that Ena/VASP proteins promote conjugate stability through actin polymerization and integrin recruitment at the immunological synapse.

**Figure 8:**
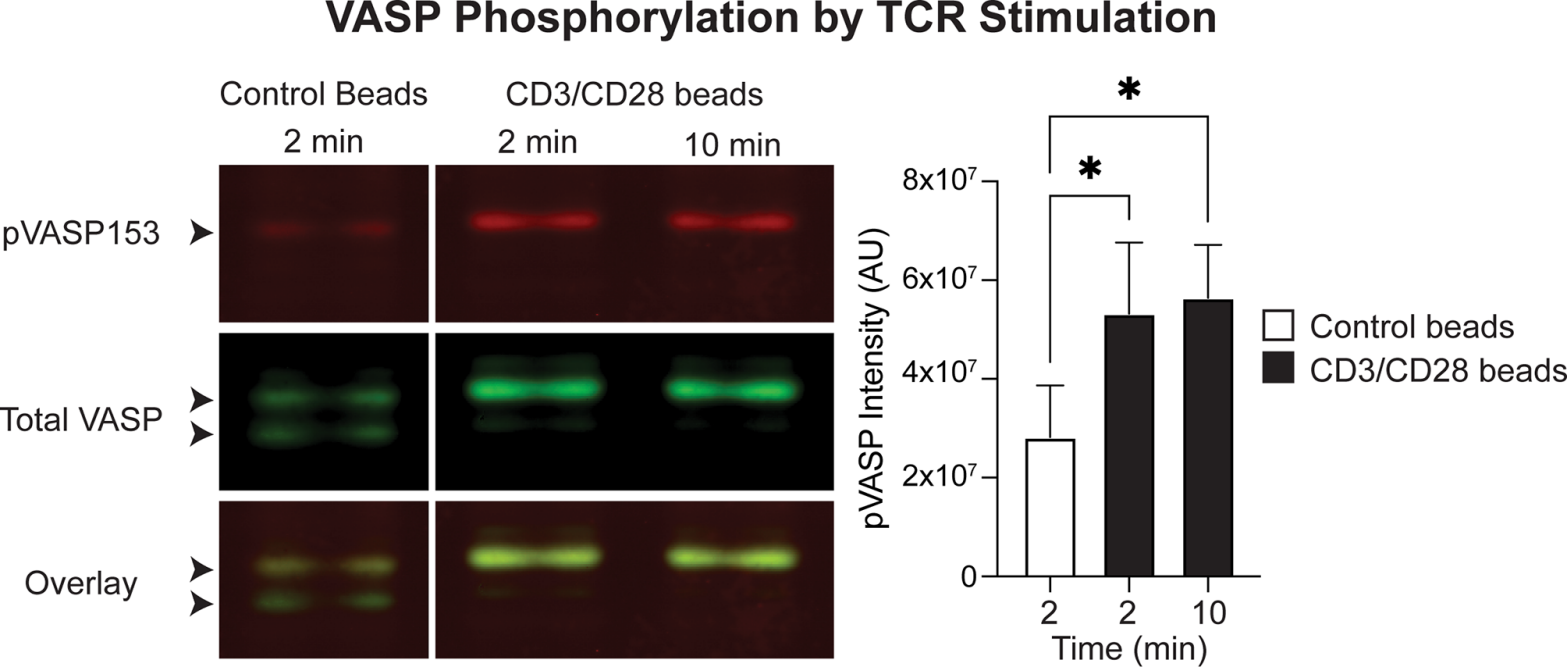
VASP is activated downstream of CD3/CD28 stimulation in T cells. *In vitro* activated T cells were stimulated with control or anti-CD3/CD28 beads and VASP phosphorylation at S153 was determined by western blotting. Left; example western blot showing detection of total VASP and pVASP-S153. Arrowheads point to the VASP protein doublet: the bottom band is the non-phosphorylated isoform, and the top band is phosphorylated. Right; quantification of pVASP signal intensity, normalized to GAPDH intensity. Data is from a total of 3 independent experiments. Statistics were performed using a one-way repeated measures ANOVA; * is p < 0.05.

EVL has been implicated in actin polymerization downstream of TCR stimulation in Jurkat cells (40), but this had not previously been assessed for VASP or in primary T cells. VASP activation is modulated by phosphorylation at multiple sites and the Serine 153 (S153) site has been shown to control subcellular localization of VASP to the cell membrane (43). Thus, to assess whether TCR engagement can activate VASP and trigger recruitment of VASP to the synapse, we next stimulated polyclonal T cells using anti-CD3/CD28 conjugated beads, and quantified VASP phosphorylation at the S153 site. Indeed, VASP S153 phosphorylation was significantly elevated after CD3/CD28 stimulation (**Figure 8**), suggesting that TCR engagement by APCs can recruit VASP to the T cell membrane for actin remodeling.

## Discussion

The goal of this work was to determine whether actin polymerization mediated by the Ena/VASP protein family plays a role in T cell priming and expansion *in vivo*. Using T cells from EVL/VASP double-knockout mice, we demonstrate that Ena/VASP proteins play an important role in the accumulation of T cells over the course of an immune response following either *L. monocytogenes* infection or subcutaneous immunization with BMDCs. We also found that the Ena/VASP family promotes efficient T cell scanning and interactions with APCs *in vivo*, resulting in fewer activated EVL/VASP dKO T cells, supporting a role for Ena/VASP proteins in optimizing T cell priming. Investigating the potential mechanisms through which EVL and VASP regulate T cell–APC interactions, we determined that T cells deficient in Ena/VASP proteins form T cell–APC conjugates with less F-actin and that are less stable than WT conjugates. Importantly, the stability and duration of T cell–APC conjugates can affect the ensuing T cell response (51, 57, 58). We thus conclude that Ena/VASP family-mediated actin polymerization downstream of TCR signaling plays an important role in the initiation and stabilization of T cell–APC conjugates, which likely drives the impaired Ena/VASP-deficient T cell accumulation phenotype.

The microenvironment of the LN is relatively permissive for migration, but an environment characterized by large numbers of tightly packed motile cells constantly exposes T cells seeking interactions with APCs to mechanical stresses. Not unlike navigating through a dense crowd with a partner at a concert, where staying still and together can be difficult, the motion of all proximal cells in a LN is interdependent and maintaining adhesive cell-cell interactions with APCs can be mechanically challenging. Furthermore, the chemokine milieu in LNs is favorable for rapid T cell motility. In fact, CCR7 signals have been demonstrated to override ‘stop signals’ from peptide-MHC *in vitro (* 59). However, CCR7 chemokines can also promote transient tethering of T cells to neighboring chemokine-coated cells, which then favors subsequent interaction with antigen-bearing APCs (60). Thus, sustained interactions with APCs must be able to overcome motility signals as well as be strong enough to withstand the physical and mechanical forces of the surrounding environment. Throughout the early phases of priming, T cells engage in both transient and sustained interactions with APCs (11, 12, 51, 55). Additionally, T cells engage in motile interactions, termed kinapses, in which T cells continue migrating over the surface of an APC (61, 62). Eventually, activation signals from TCR and costimulatory receptors accumulate over time and serve as ‘stop signals’ by inducing cellular changes (13), mediated primarily by actin rearrangement, necessary to establish tight binding and sustained T cell–APC interactions. For example, actin polymerization mediated by PKCθ inhibition and WASp activation favors stable IS formation over migration (63, 64). High-affinity antigen signals induce actin-related protein 2/3 complex (Arp2/3) activity, which has also been shown to promote arrest *in vitro (* 65). Furthermore, formin-mediated actin flow sweeps the integrin LFA-1 towards the center of the immune synapse, activating and stabilizing its high affinity conformation to enable tight binding to ICAM-1 on APCs, which controls the stability and duration of T cell–APC contacts (21, 22, 66–68).

Based on our observations, EVL/VASP-deficient T cells interact less effectively with APCs *in vivo* during initial phases of T cell priming. Given that homeostatic intranodal motility is normal in naïve EVL/VASP dKO T cells, this defect does not stem from the inability to cover sufficient ground in the LN, but instead is likely an impairment in a T cell’s ability to stop and to form or maintain interactions with cognate APCs. Studies in Natural Killer (NK) cells identified a role for EVL in the generation of F-actin at the cytotoxic synapse impacting NK cell–target cell adhesion *in vitro (* 69). In T cells, multiple molecules that have been implicated in maintaining the stability of T cell–APC interactions, including the actin bundling protein L-Plastin and ADAP, ultimately play roles in the organization, localization, and stability of LFA-1 (67, 68, 70). We have previously demonstrated a role for Ena/VASP proteins in the expression and function of the α_4_ integrin (CD49d) in T cells (49). Additionally, VASP has been associated with integrin activation in other cell types, including α_IIb_β_3_ activation in platelets (71). Thus, we assessed F-actin and LFA-1 enrichment at the immunological synapse in EVL/VASP dKO T cells. We show that EVL/VASP dKO T cells exhibit impaired actin polymerization and a small but significant reduction of LFA-1 localization at the IS. This suggests that EVL/VASP proteins promote conjugate stability through actin polymerization and integrin recruitment. Nevertheless, future studies should more closely explore the role of EVL/VASP proteins in LFA-1 clustering and stability at the IS.

During the first few hours of T cell interactions with antigen-bearing DCs *in vivo*, T cells progressively receive activation signals that can lead to CD69 upregulation (11, 12). We observed fewer EVL/VASP dKO T cells expressing CD69 after initial priming, which supports our observation that the EVL/VASP dKO T cells interact less effectively with APCs. Furthermore, it has been demonstrated that biasing towards transient interactions and signaling, as opposed to prolonged contacts, can lead to abortive T cell activation (58, 72). Our quantification of non-productive T cell–APC contacts (lasting less than 2 min) showed that EVL/VASP dKO T cells have an increased rate of aborted T cell interactions. It is possible that over time, most EVL/VASP dKO T cells will form enough interactions with APCs to trigger the start of activation, but having a higher frequency of non-productive interactions with APCs, their activation signals may not be sufficient for a robust proliferative response *in vivo*. Our *in vitro* proliferation data suggest that EVL/VASP-deficient T cells can proliferate normally. Thus, the reduced number of EVL/VASP dKO T cells is not related to intrinsic defects in cell division, but likely compounds from reduced T cell accumulation over time in the *in vivo* setting due to inefficient stimulation. Given that Ena/VASP-deficient T cell proliferation is already impaired by 72 hours after stimulation, these deficits can intensify over time and contribute to the significant reduction of EVL/VASP dKO T cells by the peak of the immune response. It will be interesting for future studies to explore the strength of activation signals in EVL/VASP dKO T cells under physiological *in vivo* conditions.

The experiments we conducted *in vitro* were used to assess whether Ena/VASP deficiency plays a role in T cell activation signals and the maintenance of interactions. In these experiments, T cell–APC contact was enforced by spinning them down together. Because previous experiments have demonstrated that T cells need approximately 2–3 minutes to “decide” whether to initiate conjugation (73), we assessed actin polymerization after 2 minutes of coculture with cognate APCs. We found clear differences in actin polymerization in EVL/VASP dKO T cells, indicating that EVL and VASP mediate actin rearrangement during T cell–APC interactions. However, the formation of conjugates, downstream signaling, and proliferation were unimpacted in these *in vitro* conditions. These data support that when T cell–APC interactions are forced by centrifugation or by high cell numbers and proximity *in vitro*, Ena/VASP proteins are dispensable for T cell activation. Thus, we posit that these *in vitro* systems lack the necessary physiological microenvironment to identify the requirement for EVL/VASP function in T cell activation. In particular, *in vitro* models are insufficient at encompassing the processes of T cell–APC scanning and interactions within tissues. However, in physiological *in vivo* conditions, we find that T cell stopping and conjugate formation with APCs is impaired by Ena/VASP deficiency. Thus, we conclude that a key function of Ena/VASP proteins in naïve T cell activation is in the establishment of T cell–APC interactions *in vivo*. Our data demonstrating that VASP is activated by CD3+CD28 stimulation and that Ena/VASP protein deficient T cell–APC conjugates are less stable than their WT counterparts further support that reduced actin polymerization and LFA-1 accumulation contribute to the EVL/VASP dKO T cell’s impaired ability to form conjugates with APCs *in vivo*.

There are still open questions regarding the specific location and contribution of Ena/VASP proteins in T cell–APC interactions and IS formation and organization. In subsequent studies, these parameters should be assessed under physiological *in vivo* conditions, and thus standard *in vitro* methods for visualizing the IS may not be appropriate. Furthermore, while this work clearly demonstrates a role for Ena/VASP proteins in mediating naïve T cell–APC interactions and activation, productive T cell–APC contacts are also essential for activated T cell functions. Thus, future studies could explore the functional implications for EVL/VASP dKO T cells in downstream effector functions and protective immunity. For example, it would be interesting to assess whether EVL/VASP dKO T cells exhibit defects in cytokine production, pathogen clearance, and memory formation.

While Ena/VASP proteins have been described to play varying roles in enhancing or suppressing motility in different cell types (35, 42, 44–46), our work is the first to explore Ena/VASP proteins in naïve T cell motility. We demonstrate that Ena/VASP proteins are dispensable for homeostatic naïve T cell intranodal migration. Ena/VASP proteins have also been implicated in regulating adhesion in other cell types (37, 40, 45), but our work uncovers a previously unidentified role for Ena/VASP proteins in stabilizing initial T cell interactions with APCs. Additionally, while EVL was suggested to play a role in actin polymerization downstream of CD3 engagement in Jurkat cells (48) and in Natural Killer cell cytotoxicity (69), our work identifies a role for Ena/VASP proteins in actin polymerization in response to antigen-bearing APCs in primary T cells. Finally, we also demonstrate a novel role for Ena/VASP proteins in promoting T cell activation and accumulation *in vivo*.

## Methods

### Mice

EVL KO mice were generated by Kwiatkowski et al. (74) and VASP KO mice were generated by Aszodi et al. (75). EVL/VASP dKO mice (originally on a 129/C57BL/6 mixed background) were a gift from Dr. Frank Gertler (MIT). The EVL/VASP dKO mice were backcrossed to C57BL/6 at least 8 times and then crossed with OT-I TCR transgenic mice (also on the C57BL/6 background). CD45.1 congenically-marked C57BL/6 recipient mice were purchased from Charles River (Strain #564). WT OT-I T cells were isolated from transgenic OT-I CD45^.1/.2^ C57BL/6 mice, and EVL/VASP-deficient OT-I T cells were isolated from transgenic EVL^-/-^ VASP^-/-^ OT-I CD45^.2/.2^ C57BL/6 mice, unless otherwise specified. This study and mouse protocol were reviewed and approved by the Institutional Animal Care and Use Committees at the University of Colorado Anschutz Medical Campus and at National Jewish Health, and all efforts were made to minimize mouse suffering.

### Bone Marrow Chimeras

Bone marrow chimeras were generated by γ-irradiating CD45^.1/.1^ congenically-marked C57BL/6 recipient mice at 500 Rads twice, four hours apart. Immediately following the second irradiation treatment, mice were reconstituted by I.V. injection of at least 1×10^6^ cells from bone marrow isolated from either transgenic OT-I CD45^.1/.2^ C57BL/6 mice or transgenic EVL^-/-^ VASP^-/-^ OT-I CD45^.2/.2^ C57BL/6 mice. Bone marrow chimeras were given at least 8 weeks to mature before use in experiments. Both WT and EVL/VASP dKO OT-I T cells reconstituted recipient mice equivalently and averaged at least 90% reconstitution by the donor cells based on congenic marker expression.

### Flow Cytometry

Activation and expansion of T cells was assessed *via* Flow Cytometry on a Fortessa flow cytometer (Beckton Dickinson) or an Aurora spectral flow cytometer (Cytek). T cell numbers were determined using CountBright Absolute Counting beads (Invitrogen, Cat. C36950) and proliferation was assessed by Cell Trace Violet (CTV, Thermo Fisher Scientific) dilution. The following antibodies/dyes were used throughout this project:

### Infection and Immunization

Congenically distinct WT (CD45^.1/.2^) and EVL/VASP dKO (CD45^.2/.2^) OT-I T cells were isolated using negative selection with an EasySep Mouse CD8^+^ T cell isolation kit (STEMCELL Technologies, Cat. 19853) from either endogenously matured or bone marrow chimera matured mice and co-transferred into CD45^.1/.1^ WT recipient mice (10,000 T cells of each for day 6 LM-OVA and BMDC time course experiments; 20,000 T cells of each for two-photon imaging experiments and day 3 harvests). To assess proliferation on day 3 in both LM-OVA and BMDC time course experiments, prior to transfer T cells were first dye-labeled with 2.5 μM CellTrace Violet in PBS for 20 minutes. 24 hours post T cell transfer, recipient mice were immunized or infected.

**Table:** 

Target	Fluorophore	Brand/catalog number
CD8α	BUV-805	BD Biosciences/612898
CD45.1	PE	Biolegend/110707
CD45.2	APC-efluor780	eBioscience/47-04540-82
KLRG1	BV-711	Biolegend/138427
CD127	BV-421	Biolegend/135023
Vα2	Alexa Fluor 647	Biolegend/127812
CD69	PE-Cy7	ThermoFisher/25-0691-82
Dead cells	Zombie Yellow	Biolegend/423104
NFATc2 (NFAT1)	Alexa Fluor 488	CST/14324S
NFκB	PE	CST/9460S
β-tubulin	Alexa Fluor 647	CST/3624S
CellTrace Violet	–	ThermoFisher/C34557
CellTrace Far Red	–	ThermoFisher/C34572
CellTrace CFSE	–	ThermoFisher/C34554
Phalloidin	Alexa Fluor 647	ThermoFisher/A22287

**Table:** 

Target	Brand/catalog number
Biotin anti-mouse CD4	Biolegend/100404
Biotin anti-mouse B220	Biolegend/103204
Biotin anti-mouse CD19	Tonbo/30-0193-U500
Biotin anti-mouse Ter119	Tonbo/30-5921-U500

For infection experiments, mice were injected with 2×10^3^ CFUs recombinant *L. monocytogenes* expressing full-length OVA (LM-OVA) and an erythromycin (Erm) resistance (ErmR) marker through the lateral tail vein (76). LM-OVA was grown and titrated as previously described (77): 1×10^8^ mouse-passaged LM-OVA aliquots were frozen at −80°C, thawed, and used to inoculate 10 mL of fresh Brain Heart Infusion (BHI) broth with Erm, grown at 37°C in a shaker overnight, then split into fresh BHI broth without Erm and grown for 2–3 hours to log phase. Titer estimates were determined by OD_600_ values, and 2×10^3^ CFU injections were prepared in PBS. Spleens were harvested 3 or 6 days after immunization for analysis by flow cytometry. T cell numbers were determined by flow cytometry, and total T cell numbers for a given tissue were calculated using CountBright Absolute Counting beads (Invitrogen, Cat. C36950). To account for potential differences in the actual T cell injection ratio and/or homing and survival of WT vs. EVL/VASP dKO T cells *in vivo*, we normalized the T cell numbers for each organ (i.e. spleen or LN) from immunized mice to the ratio of dKO/WT T cells in the equivalent organ and timepoint from non-immunized mice. Specifically, to normalize, we divided the EVL/VASP dKO numbers in the immunized organ by the dKO/WT ratio in the respective unimmunized organ.

For footpad immunizations, tdTomato-BMDCs were generated by culturing bone marrow from 3.5–8 week old B6.Cg-Gt(ROSA)26Sortm14(CAG-tdTomato)Hze/J (Rosa-Red mice) mice (Jackson Strain #007914) (in which the stop cassette was floxed-out in the germline) for 7–9 days in the presence of GMCSF. IL-4 was added to cultures to mature DCs 48 hours prior to use. Purity was typically more than 95%. BMDCs were pulsed and activated with 2 ng/mL OVA_257–264_ peptide and 1 mg/mL LPS for 1 hour and washed 3x before subcutaneous (s.c.) injection. Mice were anesthetized with isoflurane, and 2.5×10^5^ mature, antigen-pulsed activated BMDCs in a total volume of 25 μL PBS were administered s.c. in the hind footpads of mice. T cell numbers in each organ were measured and analyzed as described above for the LM experiments. OVA_257–264_ peptide (SIINFEKL) was purchased from Chi Scientific or Genscript and LPS was purchased from Invitrogen (Cat. 00-4976-93). GMCSF and IL-4 were made in house using G6 and I3L6 cell lines respectively (a gift of Dr. Matthew Krummel, UCSF).

### Isolation of Cells

For isolation of T cells from draining LNs or spleens at timepoints 72 hours or less after immunization, footpad immunized and LM-OVA infected mice were first euthanized by CO_2_ at the indicated time points, and the draining popliteal LNs and spleens were then removed and minced in 500 μL HBSS (Life Technologies) +10% FBS containing 1 mg/mL Collagenase D (Roche) and 50 μg/mL DNase I (Worthington Biochemical) per LN to break up T cell–APC conjugates. After a 30 minute incubation at 37°C, one volume of 0.1 M EDTA was added, and cells were incubated 5 additional minutes. Dissociated cells were then washed with HBSS containing 5 mM EDTA and forced through 70 μm strainers to generate single cell suspensions. After digestion, T cells from LM-OVA infected spleens at day 3 were enriched by negative selection using the following antibodies (see table below) and streptavidin beads (Biolegend, Cat. 480016). Finally, the remaining splenocytes were stained for flow cytometry analysis as above.

For isolation of T cells from the spleen and LNs of immunized and infected mice at later time points, organs were harvested dissociated and forced through 70 μm strainers to generate single cell suspensions. For collection of T cells in the blood, ~1 mL of blood was collected by cardiac puncture immediately after euthanasia and RBCs were lysed in 175 mM NH_4_Cl.

### *In Vitro* Activation and Proliferation

Spleens from C57BL/6 mice were harvested and red blood cells lysed for 2.5 minutes in 175 mM NH_4_Cl. Splenocytes were pulsed with various concentrations of SIINFEKL for 30 minutes, and washed 3 times. WT and EVL/VASP dKO OT-I T cells were isolated from lymphocytes *via* negative selection with a CD8^+^ EasySep kit. OT-I T cells were dye-labeled with CTV (1.67 μM in PBS) for 20 minutes, quenched with FBS for 5 minutes and washed 2 times. WT and EVL/VASP dKO OT-I T cells were plated in separate wells with peptide-pulsed splenocytes at a 1:3 ratio in 100 μL in a round bottom 96 well plate. Cells were fed at 48 hours by adding to each well 1 volume of fresh media and 10 U/mL IL-2 (obtained through the AIDS Research and Reference Reagent Program, Division of AIDS, NIAID, NIH from Dr. Maurice Gately, Hoffmann - La Roche Inc.). On day 3, cells were analyzed by flow cytometry. FlowJo’s (Beckton Dickinson) proliferation tool was used to analyze CTV dilution and quantify % of cells divided.

### Nucleus Flow Cytometry

Nuclei isolation and flow cytometry staining of OT-I T cells stimulated in cocultures were performed as previously described (78). Briefly, WT and EVL/VASP dKO OT-I CD8^+^ T cells were negatively enriched from LNs and spleens (using CD8^+^ EasySep kits) and labeled with 2.5 μM CTV dye for 15 minutes prior to mixing with peptide-pulsed splenocytes at a 3:1 ratio of APCs to T cells. Cells were briefly/gently spun together in FACS tubes (214 g for 1 minute) and cocultured for 45 minutes at 37°C with 10% CO_2_. For nuclei isolation, cells were treated and washed with sucrose and detergent buffers (78). The nuclei were fixed in 4% paraformaldehyde, and then intranuclear staining was performed with a 0.3% Triton-X 100 detergent PBS buffer.

### *In Vivo* Two-Photon Microscopy

CD8^+^ T cells were isolated from WT OT-I or EVL/VASP dKO OT-I mice (using CD8^+^ EasySep kits), labelled for 15–25 minutes at 37°C with 2.5 μM CTV or 0.2 μM CellTrace Far Red (CTFR, Thermo Fisher) and 20×10^3^ of each WT and EVL/VASP dKO OT-I T cells were transferred into WT recipient mice by injection into the tail vein. Fluorescent dyes were swapped between WT and EVL/VASP dKO T cells between experimental repeats to control for potential effects from the dyes. In a subset of experiments, 200×10^3^ polyclonal WT T cells dye-labelled with Carboxyfluorescein succinimidyl ester (CFSE) were also co-transferred with the OT-I T cells as a negative control. The following day, tdTomato-BMDCs were pulsed with 2 ng/mL OVA_257–264_ and activated with 1 μg/mL LPS. 2.5×10^5^ BMDCs in 25 μL PBS were injected into the hind footpads of recipient mice. Between 13–19 hours following immunization, mice were euthanized and their draining popliteal LNs were surgically removed for imaging [similar to (79)]. Explanted LNs were immobilized on coverslips with the efferent lymphatics adhered to the coverslip with Vetbond (3M). Mounted LNs were positioned on a heated flow chamber from Warner instruments (PH-1). During imaging, LNs were maintained at 35–37°C in a flow chamber perfused with RPMI medium without phenol red (Gibco) saturated with 95% O_2_/5% CO_2_. Two-photon imaging was done using a Leica SP8 DIVE upright two-photon microscope with a SpectraPhysics InsightX3 dual line (tunable 680–1300 nm and 1045 nm) IR laser with pre-chirp compensation, 4 tunable non-descanned detectors, galvanometer confocal scanner and high-speed resonant confocal scanner. Time-lapse image acquisition was done by repeated imaging of XY planes of 512x512 pixels at 1.16 μm/pixel and Z-steps of 3 μm with XYZ stacks acquired every 30–60 sec for 30–60 min.

### Two-Photon Image Analysis

Image analysis was performed using Imaris (Bitplane) and MATLAB (MathWorks). Images were linearly unmixed for possible bleed-over between channels, as previously described (80). Cells in lymph nodes that could be tracked for ≥10 minutes were used to obtain mean square displacement (MSD), speed, and arrest coefficient (time spent migrating at <2 µm/min). The Imaris ‘surface’ function was used to create a volume rendering of the T cells and BMDCs. T cell and BMDC motility was then tracked and T cell–APC interaction frequency and duration were calculated with a custom MATLAB script using a maximum distance between cells of 1 pixel (1.16 µm), with a minimum interaction of two minutes to be defined as an interaction.

### Conjugate Assays and Actin Polymerization

CD8^+^ T cells were negatively enriched from OT-I spleens (using CD8^+^ EasySep kits) and labeled with 2.5 μM CTV in PBS for 15 min. For APCs, spleens were harvested from CD45^.1/.1^ WT mice, RBCs lysed for 2.5 minutes in 175 mM NH_4_Cl, and the splenocytes dye-labeled with 1 μM CFSE in PBS for 15 minutes. Splenocytes were pulsed with various concentrations of OVA peptide for 30 minutes, washed, and mixed with T cells at a 3:1 ratio of APCs to T cells (600,000:200,000 in 100 μL). Cells were briefly spun together in FACS tubes (214 g for 1 minute) and cocultured at 37°C with 10% CO_2_. After 2 minutes, cells were fixed with 2% (w/v) paraformaldehyde (Electron Microscopy Sciences) in PBS for 10 minutes. The percentage of T cells that formed conjugates with APCs was then measured by flow cytometry. For actin polymerization analysis, cells were subsequently permeabilized with 0.5% (w/v) saponin (Millipore-Sigma) and stained with phalloidin-Alexa647 (ThermoFisher, Cat. A22287). Actin polymerization was assessed by flow cytometry and ImageStream (see next section). Fold change in phalloidin staining for both WT and EVL/VASP dKO T cell conjugates was calculated by normalizing phalloidin GMFI to respective conjugates that formed in the absence of antigen (“0 pM”). For conjugate stability experiments, splenocytes were pulsed with 1000 pM OVA peptide, and after a 2 minute incubation, T cell–APC co-cultures were vortexed (at a speed of 7/10 on a Vortex-Genie2 by Scientific Industries) for 1 second before adding PFA (2%) to fix conjugates. The change in frequency of T cell–APC conjugates was then measured by flow cytometry.

### ImageStream Analysis

Cells from the same samples analyzed by flow cytometry in the conjugate assays and actin polymerization section were analyzed by imaging flow cytometry. Data was acquired and analyzed similarly to (81). In brief, images were collected on an ImageStream X cytometer for brightfield (BF), side scatter (SSC), CTV, CFSE, and Phalloidin-AlexaFluor-647 fluorescence. The gating strategy for analysis involved first selecting focused cells, *via* the ‘gradient RMS’ of the BF image, then an aspect ratio to include only doublet events. To refine the selection further, we then gated on CFSE and CTV double-positive doublets. Doublet-only T cell–APC conjugates were successfully identified using this strategy. Single color controls were used to create a compensation matrix that was applied to all sample files. T cells and APCs were defined using ‘object’ masks and identified based on fluorescence of the CFSE label (APCs) and the CTV label (T cells). After defining these objects, the interface feature was used to generate the masked region of overlap between the T cell and APC, considered the “synapse”, in which phalloidin intensity was measured as intensity at the synapse/area of the synapse.

### VASP Phosphorylation

Polyclonal WT T cells were harvested from C57BL/6 mice and activated with plate-bound anti-CD3 (2C11) and soluble anti-CD28 (PV-1) (BioXCell, Cat. BE0001-1 and BE0015) with autologous splenocytes for two days. Cells were then removed and re-plated with 10U/mL recombinant human IL-2, with media replacement every 2 days post-activation. On day 4 post-activation, dead cells were removed from the culture using a Histopaque-1119 (Sigma) density gradient. To measure VASP phosphorylation, day 7 polyclonal activated T cells were resuspended at 10×10^6^ cells/mL in serum-free RPMI supplemented with 2% BSA (Sigma) and HEPES buffer (Corning). Control non-stimulating beads were prepared using latex beads (Invitrogen, Cat. S37227) coated with 4 μg/mL polyclonal Armenian hamster IgG (BioXCell, Cat. BE0091). DynaBeads Mouse T-Activator CD3/CD28 (ThermoFisher, Cat. 11452D) were used as directed by the manufacturer for stimulations. 1×10^6^ cells were stimulated per sample by adding prewarmed beads to chilled cells, then briefly spun for 30 s at 6000 g to bring the cells and beads into contact. The samples were then placed on a 37°C heat block for 2 minutes or 10 minutes. Stimulation was quenched with cold PBS, and samples were vortexed, pelleted, and then lysed in a 1% Triton-X100 (Sigma, Cat. T9284) buffer containing Halt Protease and Phosphatase Inhibitors (Thermo Scientific, Cat. 78440). Lysates were stored at -80°C before processing. Lysates were run on SDS-PAGE with reducing buffer, then proteins were transferred to a nitrocellulose membrane. Total VASP was detected using a monoclonal rabbit antibody (Sigma; HPA005724) and pVASP-S153 (equivalent to human pVASP-S157) was detected using a monoclonal mouse antibody (Santa Cruz, Cat. sc-365564). As a loading control, GAPDH was detected using a monoclonal mouse antibody (Santa Cruz, Cat. sc-365062). For secondary antibodies, we used fluorophore conjugated donkey anti-mouse (Licor, Cat. 926-68072) and donkey anti-rabbit antibodies (Licor, Cat. 926-32213) and imaged and quantified the membranes using the Azure Biosystems Sapphire Imager and associated software.

### Conjugate Immunofluorescence

tdTomato-BMDCs were differentiated, matured, and activated as described in the “Immunization and infection” Methods section above. On day 9 of culture, BMDCs were pulsed and activated with 2ng/mL OVA_257–264_ peptide and 1 mg/mL LPS for 1 hour and washed. Nunc Lab-Tek II Chambered Coverglass (ThermoFisher, Cat. 155409) were coated with Poly-L-Lysine for 1 hour before 100,000 antigen-pulsed and activated BMDCs were plated and incubated at 37°C to enable adherence. After 1 hour, 150,000 CD8^+^ T cells isolated from WT OT-I or EVL/VASP dKO OT-I mice (using CD8^+^ EasySep kits) were gently added to chamber slides and incubated for 45 minutes to form conjugates. Cells were then fixed with 0.5% PFA for 10 minutes and stained using anti-mouse CD11a antibody (Biolegend, Cat. 101117) followed by anti-rat FITC or A647 (Jackson Immuno) and anti-mouse CD3ε A647 or FITC (Biolegend). Images were acquired on a Zeiss LSM800 scanning confocal with a 63X oil immersion objective. Image analysis was performed in ImageJ (version 2.1.0/1.53c; NIH) on unadjusted images. Receptor polarization was analyzed by defining the mean fluorescence intensity (MFI) of the contact region at the synapse divided by the MFI of a similar sized region at the back of the cell.

### Statistical Analyses

Graphs of data and statistical analyses were done using Prism software (versions 7–9, GraphPad). The individual statistical tests used to analyze each experiment and the experimental repeats and sample sizes are reported in the figure legends.

## Data Availability Statement

The original contributions presented in the study are included in the article/**Supplementary Material**. Further inquiries can be directed to the corresponding author.

## Ethics Statement

The animal study was reviewed and approved by Institutional Animal Care and Use Committee at the University of Colorado Anschutz Medical Campus and Institutional Animal Care and Use Committee at National Jewish Health.

## Author Contributions

MW designed research, performed experiments, analyzed the data, and wrote the manuscript. JR ran ImageStream and analyzed the respective data. AS helped with MATLAB script analysis and performed the experiments analyzing VASP phosphorylation. BW helped with listeria infections. JC helped with BMDC and bone marrow chimera generation. RK provided guidance and lab space for listeria experiments. RF provided experimental design guidance and feedback on the manuscript. JJ designed and supervised the research, acquired funding, participated in data analysis, and wrote the manuscript. All authors contributed to the article and approved the submitted version.

## Funding

This work was supported in part by grants from: NIAID R01AI125553 (JJ) and NIDDK R01DK111733 (RSF). MW and AS were supported in part by NIH Training Grant T32AI007405. This work was also supported in part by the University of Colorado Diabetes Research Center (DRC) grant P30DK116073; the spectral flow cytometer and the two-photon microscope employed for imaging are part of the Cell and Tissue Analysis Core of the University of Colorado Diabetes Research Center. The content of this work is solely the responsibility of the authors and does not necessarily represent the official views of the NIH.

## Conflict of Interest

The authors declare that the research was conducted in the absence of any commercial or financial relationships that could be construed as a potential conflict of interest.

## Publisher’s Note

All claims expressed in this article are solely those of the authors and do not necessarily represent those of their affiliated organizations, or those of the publisher, the editors and the reviewers. Any product that may be evaluated in this article, or claim that may be made by its manufacturer, is not guaranteed or endorsed by the publisher.

## Supplementary Material

Supplementary Figure 1 EVL/VASP dKO T cells from bone marrow chimeras are functionally the same as those isolated directly from EVL/VASP dKO mice. Bone marrow chimeras were generated by reconstituting WT CD45^.1/.1^ mice with EVL/VASP dKO OT-I CD45^.2/.2^ or WT OT-I CD45^.1/.2^ bone marrow. CD8^+^ T cells were isolated from chimeric mice ≥8 weeks after reconstitution and 10,000 of each were transferred into WT CD45^.1/.1^ recipient mice. Recipient mice were then infected I.V. with LM-OVA (2×10^3^ PFUs) the following day and analyzed for Ag-specific CD8^+^ T cell responses 6 days later. Endogenously matured CD8^+^ T cells were harvested directly from EVL/VASP dKO OT-I CD45^.2/.2^ or WT OT-I CD45^.1/.2^ mice and transferred into WT CD45^.1/.1^ recipient mice and infected in the same way. T cell numbers were normalized to the ratio of transferred EVL/VASP dKO/WT T cells recovered from spleens of uninfected recipient mice at the same timepoint. **(A)** Graphical schematic of the experimental method for obtaining donor T cells. **(B)** T cell numbers in the spleen of recipient mice at day 6 post LM infection. Recipient mice were transferred with endogenously matured T cells (left) or with bone marrow chimera matured T cells (middle). Ratios of EVL/VASP dKO/WT OT-I T cells for the two kinds of donor T cells (right panel). Each dot represents an average from a single experiment with ≥2 mice per group, from ≥4 experiments. Significance was assessed by paired t tests or for the EVL/VASP dKO/WT ratio one sample t tests compared to a hypothetical value of 1.0; ns is not significant, ** is p < 0.01, *** is p < 0.001, and **** is p < 0.0001.

Supplementary Figure 2 EVL/VASP dKO T cells navigate the LN normally under homeostatic conditions. WT OT-I and EVL/VASP dKO OT-I T cells were isolated from donor mice and differentially dye-labeled with CTV or CTFR, and 5×10^5^-1×10^6^ of each were co-transferred at a 1:1 ratio into WT recipient mice. 24 hours later, popliteal LNs were harvested and analyzed by time-lapse two-photon microscopy. **(A)** Representative snapshot from a movie depicting the movement of EVL/VASP dKO OT-I T cells (cyan) and WT OT-I T cells (red) in the absence of cognate antigen. Track lines show the path of T cell movement imaged over 10 minutes. Time in min:sec; scale bar, depicted under the time-stamp, is 20 µm. **(B)** Mean square displacement (MSD ± SEM) over time of WT and EVL/VASP dKO OT-I T cells during homeostatic conditions in the absence of cognate antigen. **(C)** Analysis of T cell motility parameters. Quantification of WT and EVL/VASP dKO OT-I T cell average mean track speed, track straightness, and arrest coefficient (percentage of each track in which instantaneous velocity <2 μm/min) during homeostatic conditions. Data represents averages from a total of 3 experiments. There was no significant difference (ns), as assessed by two-way ANOVA interaction effects **(B)** and paired T tests **(C)**.

Supplementary Movie 1 Polyclonal WT and EVL/VASP dKO T cells were isolated, differentially dye-labeled with CTV or CTFR, and co-transferred at a 1:1 ratio into WT recipient mice. 24 hours later, popliteal LNs were harvested and analyzed by time-lapse two-photon microscopy. Individual EVL/VASP dKO T cells (cyan) and WT T cells (red) were tracked in the absence of cognate antigen over 30 minutes. Track lines show the path of T cell movement imaged over the last 10 minutes. Scale bar, 20 µm.

Supplementary Movie 2 WT OT-I, EVL/VASP dKO OT-I, and polyclonal WT T cells were isolated, differentially dye-labeled, and co-transferred into WT recipient mice. 24 hours later, 2.5×10^5^ mature, LPS-activated OVA-pulsed tdTomato-BMDCs were injected subcutaneously into both hind footpads of recipient mice. After 13 hours, mice were euthanized and the draining popliteal LNs were harvested and analyzed by time-lapse two-photon microscopy. Individual T cells were tracked and their interactions with BMDCs were also quantified. This example movie depicts the movement of EVL/VASP dKO OT-I T cells (green), WT OT-I T cells (cyan), polyclonal control T cells (yellow), and pulsed BMDCs (red). Track lines show the path of T cell movement imaged over 33 minutes. Scale bar, 50 µm; time in min:sec.

Supplementary Movie 3 WT OT-I, EVL/VASP dKO OT-I, and polyclonal WT T cells were isolated, differentially dye-labeled, and co-transferred into WT recipient mice. 24 hours later, 2.5×10^5^ mature, LPS-activated OVA-pulsed tdTomato-BMDCs were injected subcutaneously into both hind footpads of recipient mice. After 13 hours, mice were euthanized and the draining popliteal LNs were harvested and analyzed by time-lapse two-photon microscopy. Individual T cells were tracked and their interactions with BMDCs were also quantified. This example movie depicts the movement of EVL/VASP dKO OT-I T cells (green), WT OT-I T cells (cyan), polyclonal control T cells (yellow), and pulsed BMDCs (red). Track lines show the path of T cell movement imaged over 30 minutes. Scale bar, 30 µm; time in min:sec.
