# Supplementary figures and images for: Ena/VASP Protein-Mediated Actin Polymerization Contributes to Naïve CD8+ T Cell Activation and Expansion by Promoting T Cell–APC Interactions In Vivo
Source: Front Immunol. 2022 Jun 9;13:856977. doi: 10.3389/fimmu.2022.856977 (PMC9222560; doi:10.3389/fimmu.2022.856977)

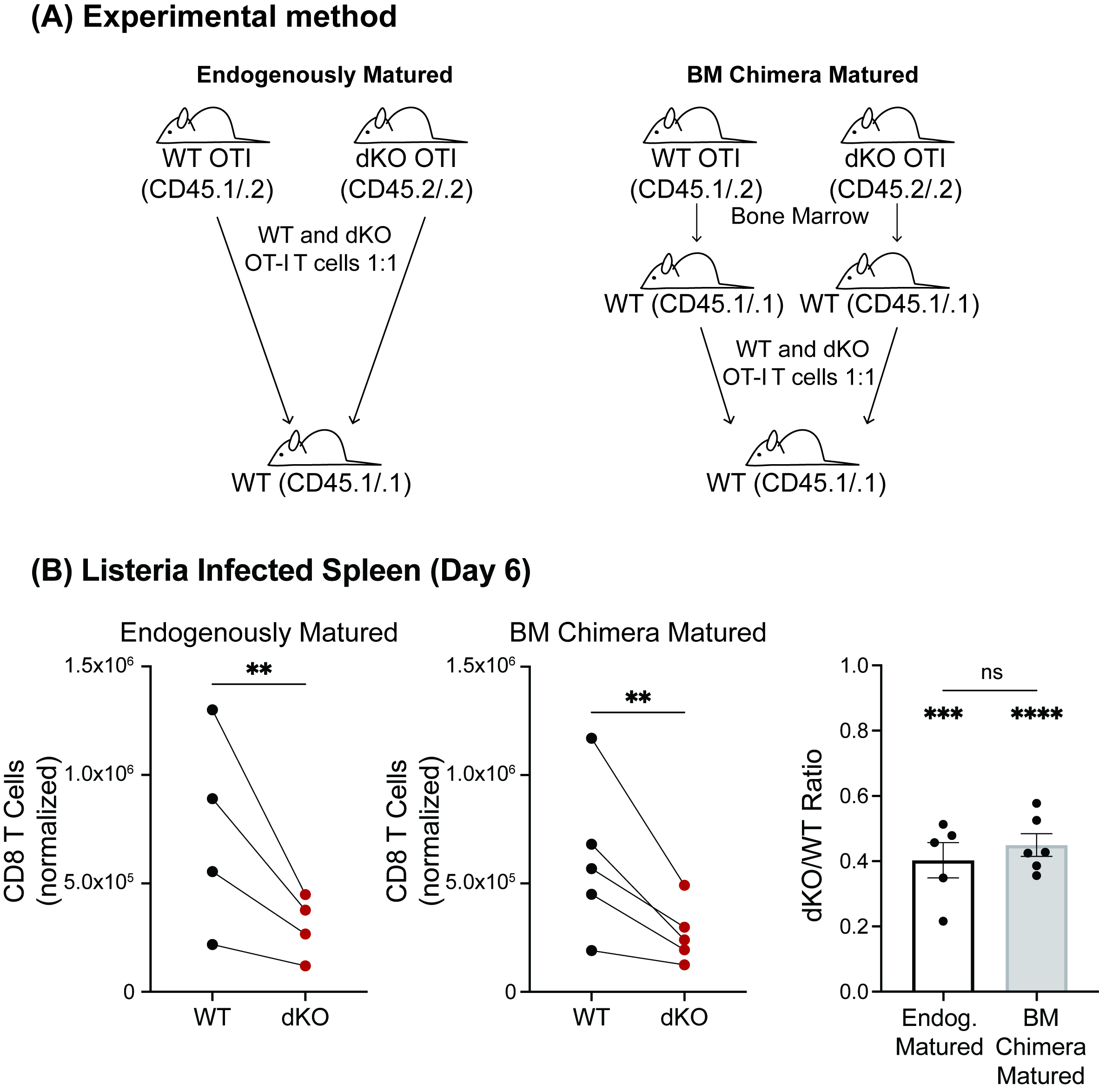

Supplement: Supplementary Figure 1 — EVL/VASP dKO T cells from bone marrow chimeras are functionally the same as those isolated directly from EVL/VASP dKO mice. Bone marrow chimeras were generated by reconstituting WT CD45.1/.1 mice with EVL/VASP dKO OT-I CD45.2/.2 or WT OT-I CD45.1/.2 bone marrow. CD8+ T cells were isolated from chimeric mice ≥8 weeks after reconstitution and 10,000 of each were transferred into WT CD45.1/.1 recipient mice. Recipient mice were then infected I.V. with LM-OVA (2×10^3^ PFUs) the following day and analyzed for Ag-specific CD8+ T cell responses 6 days later. Endogenously matured CD8+ T cells were harvested directly from EVL/VASP dKO OT-I CD45.2/.2 or WT OT-I CD45.1/.2 mice and transferred into WT CD45.1/.1 recipient mice and infected in the same way. T cell numbers were normalized to the ratio of transferred EVL/VASP dKO/WT T cells recovered from spleens of uninfected recipient mice at the same timepoint. (A) Graphical schematic of the experimental method for obtaining donor T cells. (B) T cell numbers in the spleen of recipient mice at day 6 post LM infection. Recipient mice were transferred with endogenously matured T cells (left) or with bone marrow chimera matured T cells (middle). Ratios of EVL/VASP dKO/WT OT-I T cells for the two kinds of donor T cells (right panel). Each dot represents an average from a single experiment with ≥2 mice per group, from ≥4 experiments. Significance was assessed by paired t tests or for the EVL/VASP dKO/WT ratio one sample t tests compared to a hypothetical value of 1.0; ns is not significant, ** is p < 0.01, *** is p < 0.001, and **** is p < 0.0001. [file Image_1.tif]

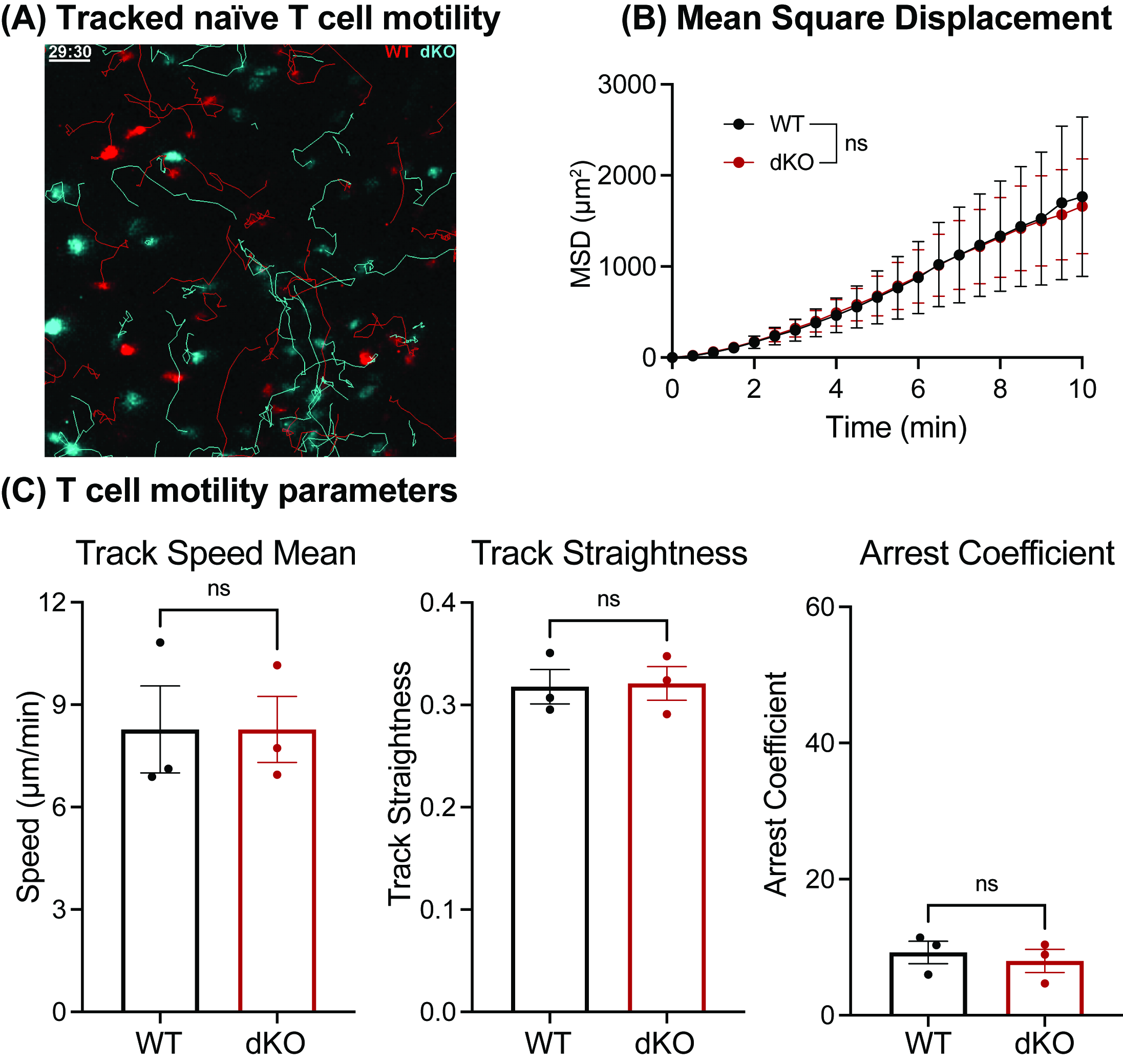

Supplement: Supplementary Figure 2 — EVL/VASP dKO T cells navigate the LN normally under homeostatic conditions. WT OT-I and EVL/VASP dKO OT-I T cells were isolated from donor mice and differentially dye-labeled with CTV or CTFR, and 5×10^5^-1×10^6^ of each were co-transferred at a 1:1 ratio into WT recipient mice. 24 hours later, popliteal LNs were harvested and analyzed by time-lapse two-photon microscopy. (A) Representative snapshot from a movie depicting the movement of EVL/VASP dKO OT-I T cells (cyan) and WT OT-I T cells (red) in the absence of cognate antigen. Track lines show the path of T cell movement imaged over 10 minutes. Time in min:sec; scale bar, depicted under the time-stamp, is 20 µm. (B) Mean square displacement (MSD ± SEM) over time of WT and EVL/VASP dKO OT-I T cells during homeostatic conditions in the absence of cognate antigen. (C) Analysis of T cell motility parameters. Quantification of WT and EVL/VASP dKO OT-I T cell average mean track speed, track straightness, and arrest coefficient (percentage of each track in which instantaneous velocity <2 μm/min) during homeostatic conditions. Data represents averages from a total of 3 experiments. There was no significant difference (ns), as assessed by two-way ANOVA interaction effects (B) and paired T tests (C). [file Image_2.tif]
